# Postoperative changes in cervical hemodynamics and cognitive function following cervical lymphatic-venous surgery in Alzheimer's disease

**DOI:** 10.1177/13872877261459037

**Published:** 2026-06-26

**Authors:** Xiaoju Zheng, Cong Tang, Haijun Li, Baoshan Wang, Yuqi Zheng, Wenbin Song, Aimei Wu, Linjun Xin, Dengwen Zhang, Rongguo Yang, Shuang Du, Peng He, Yujiao Li, Linjuan Wu, Qi Shi, Gonçalo J.L. Bernardes

**Affiliations:** 1 Xi'an Fengcheng Hospital, Xi'an, Shaanxi, China; 2 GIMM—Gulbenkian Institute for Molecular Medicine, Lisboa, Portugal; 3 Nova School of Business and Economics, Carcavelos, Portugal; 4 Yusuf Hamied Department of Chemistry, 2152 University of Cambridge, Cambridge, UK

**Keywords:** Alzheimer's disease, carotid sheath, cerebral perfusion, cognitive function, deep cervical lymphatic-venous anastomosis, hemodynamics

## Abstract

**Background:**

Alzheimer's disease (AD) has been increasingly linked to cervical venous and lymphatic dysfunction, impairing cerebral perfusion and clearance of neurotoxic metabolites. Deep cervical lymphatic-venous anastomosis (dcLVA) is a microsurgical approach aimed at restoring lymphatic-venous communication.

**Objective:**

To evaluate postoperative changes in cervical hemodynamics and cognitive function in patients with AD.

**Methods:**

Eighty-three AD patients underwent bilateral cervical LVA. Pre- and postoperative evaluations included Doppler ultrasound of the internal jugular veins and carotid arteries at both hyoid and cricoid levels, along with standardized cognitive and functional tests. Morphologic and hemodynamic changes were analyzed using the Wilcoxon and Spearman analysis.

**Results:**

Postoperatively, significant increases were observed in venous and arterial flow, along with enlargement of internal jugular and carotid cross-sectional areas. Postoperative improvements were observed, with postoperative carotid flow moderately correlated with Mini-Mental State Examination and activities of daily living. Longitudinal follow-up demonstrated sustained neuropsychological improvement up to 9 months, with persistent enhancement of vascular flow in a follow-up subgroup.

**Conclusions:**

The procedure was associated with changes in vascular parameters and clinical outcomes; however, the specific contribution of dcLVA cannot be determined.

## Introduction

The prevalence of dementia continues to rise globally, imposing growing social and economic burdens on aging populations. Alzheimer's disease (AD) is the most common form of dementia, accounting for approximately 60–70% of all cases.^1^ It is a chronic, progressive neurodegenerative disorder characterized by gradual cognitive decline, behavioral disturbances, and impaired daily functioning. The pathological hallmarks of AD include extracellular deposition of amyloid-β (Aβ) plaques and intracellular neurofibrillary tangles composed of hyperphosphorylated tau protein, leading to neuronal dysfunction and loss.^2,3^ Current pharmacologic therapies, such as cholinesterase inhibitors, NMDA receptor antagonists, and anti-amyloid monoclonal antibodies, offer only modest symptomatic benefit and do not effectively halt disease progression.^4,5^

Beyond classical amyloid and tau hypotheses, recent discoveries have reshaped our understanding of AD pathophysiology. In 2015, Louveau et al. identified functional meningeal lymphatic vessels that drain cerebrospinal fluid (CSF) and interstitial solutes, including Aβ, into the deep cervical lymph nodes, thereby establishing a direct lymph venous interface for cerebral waste clearance.^6,7^ Impaired lymphatic drainage has been linked to neuroinflammation, amyloid accumulation, and cognitive decline.^8–10^ These findings highlight the potential importance of the cervical lymphatic and venous systems in maintaining cerebral homeostasis. At the same time, recent systematic reviews and meta-analyses have shown that AD patients generally have impaired cerebral hemodynamics, including decreased mean cerebral arterial flow velocity and weakened vascular reactivity, suggesting that cerebral hypoperfusion plays an important role in the occurrence and progression of AD.^11^ Continuous hypoperfusion can lead to neuronal metabolic disorders and disruption of brain microenvironment homeostasis, thereby promoting pathological protein aggregation. At the same time, the interaction between Aβ and neurovascular dysfunction is considered to be a key link in the pathological development of AD: endothelial cell damage, decreased blood-brain barrier function, and local blood flow disorders not only accelerate Aβ deposition, but also further weaken the oxygen metabolism and nutrient supply of brain tissue, forming a vicious cycle.^12^ In this context, restoring brain fluid dynamics, especially improving the drainage function of the cerebral lymphatic and glymphatic systems, thereby promoting the clearance of metabolites and improving cerebral blood supply, may become a new way to intervene in the course of AD.

Lymphatic-venous anastomosis (LVA), a microsurgical procedure originally developed to treat lymphedema, offers a minimally invasive means to re-establish lymphatic drainage and relieve perivascular compression. A recent prospective clinical study demonstrated that deep cervical lymphatico-venous anastomosis (dcLVA) has been proposed as a potential intervention to improve cognitive and functional outcomes in patients with AD, likely through hemodynamic and lymphatic modulation.^13^ Nevertheless, the detailed vascular mechanisms underlying these improvements remain poorly understood.

From May 2024 to May 2025, our team at the Department of Hand and Microsurgery, Xi’an Fengcheng Hospital, performed lymphatic or lymph node–jugular vein anastomoses in 150 elderly patients with cognitive impairment. Among these, 83 patients underwent standardized preoperative and postoperative imaging and neuropsychological evaluation. This study aims to quantify postoperative changes in cervical vascular morphology and flow, and to assess their correlation with cognitive, functional, and behavioral outcomes following the surgical procedure in patients with AD. To our knowledge, this is the first systematic evaluation of cerebrovascular and cognitive outcomes after dcLVA in this population.

## Methods

### Study design and ethical approval

This was a prospective observational cohort study conducted at the Department of Hand and Microsurgery, Xi’an Fengcheng Hospital, from May 2024 to May 2025. The study was approved by the institutional Ethics Committee of Xi’an Fengcheng Hospital (Approval No. JS2024-001-01) and conducted in accordance with the Declaration of Helsinki. Written informed consent for both surgery and study participation was obtained from all patients or their legal guardians prior to enrollment. All dcLVA procedures were performed before the July 2025 prohibition issued by the National Health Commission. No operations occurred after the ban, and the study was designed for scientific exploration rather than clinical treatment.

### Patient selection and demographics

During the study period, a total of 150 patients underwent dcLVA surgery. Of these, 83 patients met the inclusion criteria and were enrolled for detailed analysis. The cohort comprised 25 males and 58 females, with a mean age of 69.54 years (range: 60–88 years). Based on the Clinical Dementia Rating (CDR) scale, 8 patients (10.7%) had mild dementia (CDR = 1), 15 (17.9%) had moderate dementia (CDR = 2), and 60 (71.4%) had severe dementia (CDR = 3).

Common comorbidities included lacunar cerebral infarction in 56 patients (67.5%), hypertension in 30 (36.1%), coronary artery disease in 11 (13.3%), and diabetes mellitus in 18 (21.7%). Chronic inflammation of the ethmoid sinus or nasopharynx was noted in 71 patients (85.5%), and 45 (54.2%) presented with urinary and/or fecal incontinence.

Baseline neuropsychological and functional assessments yielded the following mean scores: Mini-Mental State Examination (MMSE) = 3.01 ± 4.45 (range: 0–20); Montreal Cognitive Assessment (MoCA) = 1.54 ± 2.26 (range: 0–20); Activities of Daily Living (ADL) = 50.87 ± 30.05 (range: 0–100); Agitated Behavior Scale (ABS) = 19.19 ± 6.68 (range: 10–38).

Inclusion criteria were as follows:

1. Age 60–90 years with a confirmed diagnosis of AD made by a tertiary hospital.
2. Neurological and cardiological evaluation confirming no severe cardiovascular or coagulation disorders.
3. MMSE score ≤ 20.
4. Informed consent and a clear request for intervention from family members.

Exclusion criteria included:

1. Unconfirmed diagnosis or presence of confounding neurological disorders.
2. Severe cardiovascular or hematological contraindications to surgery.
3. Unrealistic expectations or refusal to comply with follow-up.

### Surgical procedure

**Figure 1.**
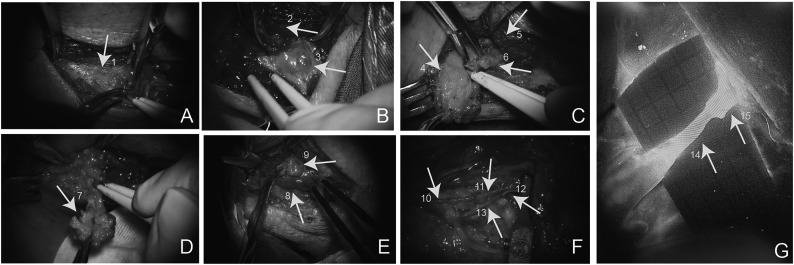
Intraoperative photographs of the deep cervical lymphatic structures and related procedures. (A) Arrow 1 indicates the external appearance of the deep cervical lymphatic sac. (B) Protruding lymphoid tissue (lymph node) labelled with arrow 3 was observed after incision of the lymphatic sac. Arrow 2 indicates the sternocleidomastoid muscle (SCM). (C) Adipose tissue mass (arrow 4) located at the jugular foramen region. Arrow 5—SCM; arrow 6—enlarged lymph nodes. (D) Removal of the adipose tissue (arrow 7). (E) Lymph node (arrow 9) compressing the IJV (arrow 8). (F) LVA performed after dissection. Arrow 10/13- subcutaneous veins; arrow 11/12- lymphatic vessels. (G) Intraoperative fluorescence ICG imaging. Arrow 14—Lymphatic vessel; arrow 15—vein.

All procedures were performed under general anesthesia by the same senior microsurgical team. Prior to incision, 0.1 ml of indocyanine green (ICG) was injected into the nasal mucosa under ultrasound guidance to visualize lymphatic drainage (Figure 1). A 5–8 cm skin incision was made along the posterior border of the platysma, extending from just below the mandibular angle to 1 cm inferior to the cricoid cartilage. After elevating the platysma, the posterior fascial layer, continuous with the deep prevertebral fascia, was exposed and opened to access the carotid sheath. Fatty tissue and lymph nodes protruded in approximately 85% of cases. Non-fluorescent or enlarged lymph nodes were excised, whereas viable candidate nodes for anastomosis were identified by their pinkish color, firm elasticity, pea-like size, and homogeneous fluorescence under ICG imaging. Appropriate tributaries of the external jugular vein were isolated for anastomosis. End-to-side lymph node–to-vein anastomosis was performed under microscopy. Where suitable, small cervical veins were used for sleeve-style or end-to-end lymphatic venous connections. The operative field was irrigated, hemostasis achieved, and a closed-suction drain placed prior to layered skin closure. The same procedure was then performed contralaterally. In cases presenting with hoarseness or dysphagia, neural decompression was performed by incising the carotid sheath to relieve perineural compression. The mean operative duration was 3.8 h (range: 2.5–8.0 h). Prior to incision, a small volume of ICG was injected into the nasal mucosa to facilitate intraoperative visualization of cervical lymphatic structures. It should be emphasized that intranasal ICG injection was used solely as a qualitative guidance method to identify functional lymphatic tissue in the cervical region and was not intended to map or quantify cerebral or meningeal lymphatic drainage pathways.

### Intraoperative pathological and morphological assessments

#### Lymph nodes

Under ICG fluorescence and microscopy, lymph nodes were identified and measured in three regions: above the hyoid (Level II), between the hyoid and cricoid (Level III), and 2 cm below the cricoid cartilage. Gross morphological characteristics were documented immediately after excision.

#### Pericarotid adipose thickness

Using a micrometer (accuracy ± 0.05 mm) under a Zeiss Tivato 700 surgical microscope, the thickness of adipose tissue between the carotid sheath and the posterior fascia of the sternocleidomastoid muscle was recorded.

#### Lymphatic vessels

Fluorescent lymphatic channels were observed and traced under ICG-enhanced microscopy.

#### Fascial and muscular structures

The sternocleidomastoid origin, posterior fascia, and adjacent fibrotic tissue between the mandibular angle and anterior muscle border were inspected for adhesions or scarring.

### Ultrasound evaluation

All sonographic assessments were performed by a single senior ultra sonographer trained in cervical vascular imaging, using a GE LOGIQ E11 ultrasound system. Flow velocity and cross-sectional area of the internal carotid artery (ICA), common carotid artery (CCA), and internal jugular vein (IJV) were measured bilaterally at the hyoid and cricoid levels.

Measurements were acquired at five standardized timepoints: before skin incision; after opening the fascial capsule; following clearance of capsule contents; after carotid sheath incision; after skin closure. For each timepoint, three consecutive measurements were taken and averaged for analysis.

Because there is currently no validated clinical method to directly quantify deep cervical lymphatic flow or to measure pressure within the deep cervical lymphatic-vascular compartment, cervical arterial and venous hemodynamic parameters were assessed as indirect surrogate indicators. Pre- and postoperative changes in blood flow and cross-sectional area were therefore used to reflect potential alterations within this confined cervical fascial space, rather than to directly measure lymphatic drainage.

### Postoperative management and follow-up

Patients were maintained in a quiet environment postoperatively and remained on bed rest for 3 h, followed by a semi-recumbent position. Oral fluids and ambulation were resumed 6 h after surgery. Standard prophylactic antibiotics were administered, and drainage color and volume were monitored.

From postoperative day 2, a structured rehabilitation program was initiated. All rehabilitation procedures were performed by certified rehabilitation therapists who had received standardized in-hospital training. Neck massage therapy was administered once daily (20 min per session). The head, face, and bilateral cervical regions were divided into standardized anatomical zones. Manual techniques including pressing, kneading, and gentle soft-tissue mobilization were applied, particularly along the sternocleidomastoid muscle and perivascular areas. Repetitive transcranial magnetic stimulation was delivered at 10 Hz to the left dorsolateral prefrontal cortex to enhance executive and memory-related cortical activity. Treatment was administered five times per week for four consecutive weeks. Patients with motor impairments underwent individualized physiotherapy programs consisting of lower limb muscle strengthening, balance training using a balance board, gait correction exercises, and passive joint range-of-motion maintenance. Sessions were conducted once daily. Patients with aphasia received one-on-one speech therapy including picture naming, sentence repetition, and structured situational dialogue training. In selected cases, transcranial direct current stimulation was applied to language-related cortical regions to enhance cortical excitability. Speech therapy was delivered five times per week.

Neuropsychological evaluations including MMSE, MoCA, ADL, and ABS were repeated postoperatively during hospitalization and at 1 month, 3 months, 6 months, and 9 months via outpatient visits, WeChat consultations, or telephone interviews, depending on patient availability. Because of attrition and logistical constraints, the number of patients available at each follow-up time point varied. In addition, a subgroup of patients (n = 9) underwent repeat cervical Doppler ultrasound at follow-up to assess longitudinal changes in vascular cross-sectional area and blood flow at the hyoid and cricoid levels.

### Statistical analysis

Statistical analyses were performed using GraphPad Prism (version 9.0, GraphPad Software, San Diego, CA, USA) and Python (version 3.11) with the SciPy (version 1.11.4; scipy.stats) and statsmodels (version 0.14.0) libraries. Continuous variables are presented as mean ± standard deviation (SD). Paired comparisons between preoperative and postoperative values were performed using the Wilcoxon signed-rank test. Associations between vascular parameters (cross-sectional area and blood flow) and clinical scores (MMSE, MoCA, ADL, and ABS) were calculated using Spearman's rank correlation coefficient. A two-sided p < 0.05 was considered statistically significant.

## Results

### Postoperative cognitive and behavioral improvements

Postoperative improvements in clinical measures were observed in this cohort. All cases (100%) exhibited enhanced alertness and vitality, characterized by brighter affect, increased responsiveness, and improved engagement after surgery typically lasting for 2–3 days. Most patients regained the ability to recognize and appropriately interact with family members, accompanied by notable recovery in limb motor function and ADL compared with preoperative performance. Attention and executive functions were also significantly improved, enabling more effective interaction with caregivers. Moreover, patients who presented with preoperative emotional disturbances, hoarseness, dysphagia with choking episodes during swallowing, or hearing impairment experienced significant relief of symptoms after surgery. Among 45 patients with preoperative urinary or fecal incontinence, 37 (82.2%) demonstrated symptom improvement, while 8 (17.8%) showed no remarkable change. This early postoperative increase in alertness and vitality was transient and is most likely related to acute autonomic or neural modulation rather than sustained cognitive recovery.

**Figure 2.**
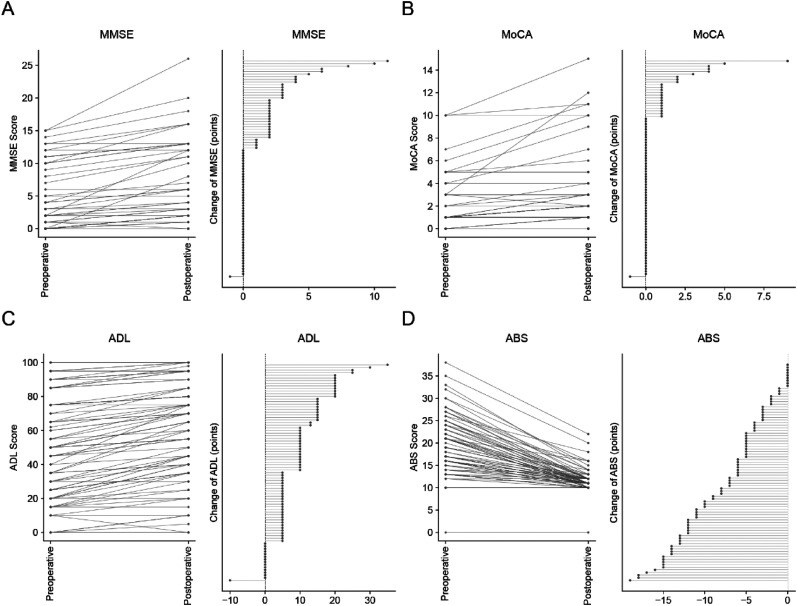
Preoperative and postoperative changes in neuropsychological and behavioral scores of 83 patients undergoing dcLVA surgery. (A) Mini-Mental State Examination (MMSE), (B) Montreal Cognitive Assessment (MoCA), (C) Activities of Daily Living (ADL), and (D) Agitation Behavior Scale (ABS) scores were assessed before and after surgery. The left panels depict individual preoperative and postoperative scores connected by lines, illustrating overall improvement trends across patients. The right panels display the distribution of individual score changes (postoperative minus preoperative). An upward shift indicates improvement in MMSE, MoCA, and ADL, while a downward shift represents a reduction in agitation severity (ABS). Statistically significant differences were observed across all domains (p < 0.0001 for all comparisons; see Table 1 for summary statistics).

All patients underwent standardized neuropsychological and behavioral evaluations both before and after surgery. As shown in Table 1 and Figure 2, statistically significant postoperative improvements were shown in cognitive, functional, and behavioral performance.

**Table 1.** Scoring and preoperative and postoperative differential tests of 83 cases.

Score	Preoperative	Postoperative	Difference (Post-Pre)	p
MMSE	3.01 ± 4.45	4.33 ± 5.9	**1.31** **±** **2.22**	0.0000
MoCA	1.54 ± 2.26	2.11 ± 3.15	**0.57** **±** **1.41**	0.0000
ADL	50.87 ± 30.05	59.92 ± 29.05	**9.05** **±** **7.82**	0.0000
Agitation behavior	19.19 ± 6.68	11.67 ± 2.53	**−7.52** **±** **5.28**	0.0000

The mean MMSE score increased from 3.01 to 4.33 (p < 0.0001), with 34 patients (40.96%) improved, 1 (1.20%) declined, and 48 (57.83% unchanged. Similarly. The mean MoCA score improved from 1.54 to 2.11 (p < 0.0001), with 22 patients (26.51%) improved, 1 (1.20%) declined, and 60 (72.29%) unchanged. The functional status, assessed using the ADL score, improved significantly from 50.87 to 59.92 (p < 0.0001), with 68 patients (81.93%) improved, 1 (1.20%) declined, and 14 (16.87%) unchanged.

Behavioral symptoms also improved significantly. The mean ABS score decreased from 19.19 to 11.67 (p < 0.0001), with 74 patients (89.15%) reduced and 9 (10.84%) unchanged. These results describe postoperative changes observed in this cohort.

Notably, one patient showed no change across all scores, whose baseline score was severely deficient (MMSE = 0, MoCA = 0, ADL = 10, ABS = 10).

### Longitudinal neuropsychological outcomes

To evaluate the durability of cognitive and functional improvement, longitudinal neuropsychological follow-up was performed at 1 day, 1 week, 1 month, 3 months, and 9 months postoperatively in available patients (sample size varied by time point). The results are summarized in Supplemental Table 2 and Supplemental Figure 1.

Compared with the preoperative baseline, statistically significant improvements were observed across all four measures (MMSE, MoCA, ADL, ABS) at each available follow-up time point (all p < 0.05). Although some patients demonstrated partial regression toward baseline at intermediate follow-up visits, the overall group-level improvement was sustained. Notably, functional (ADL) and behavioral (ABS) improvements remained stable through 9 months.

These findings suggest that the neuropsychological benefits of the surgery are not limited to an acute perioperative effect but demonstrate persistence over mid-term follow-up.

### Postoperative changes in vascular cross-sectional area

**Figure 3.**
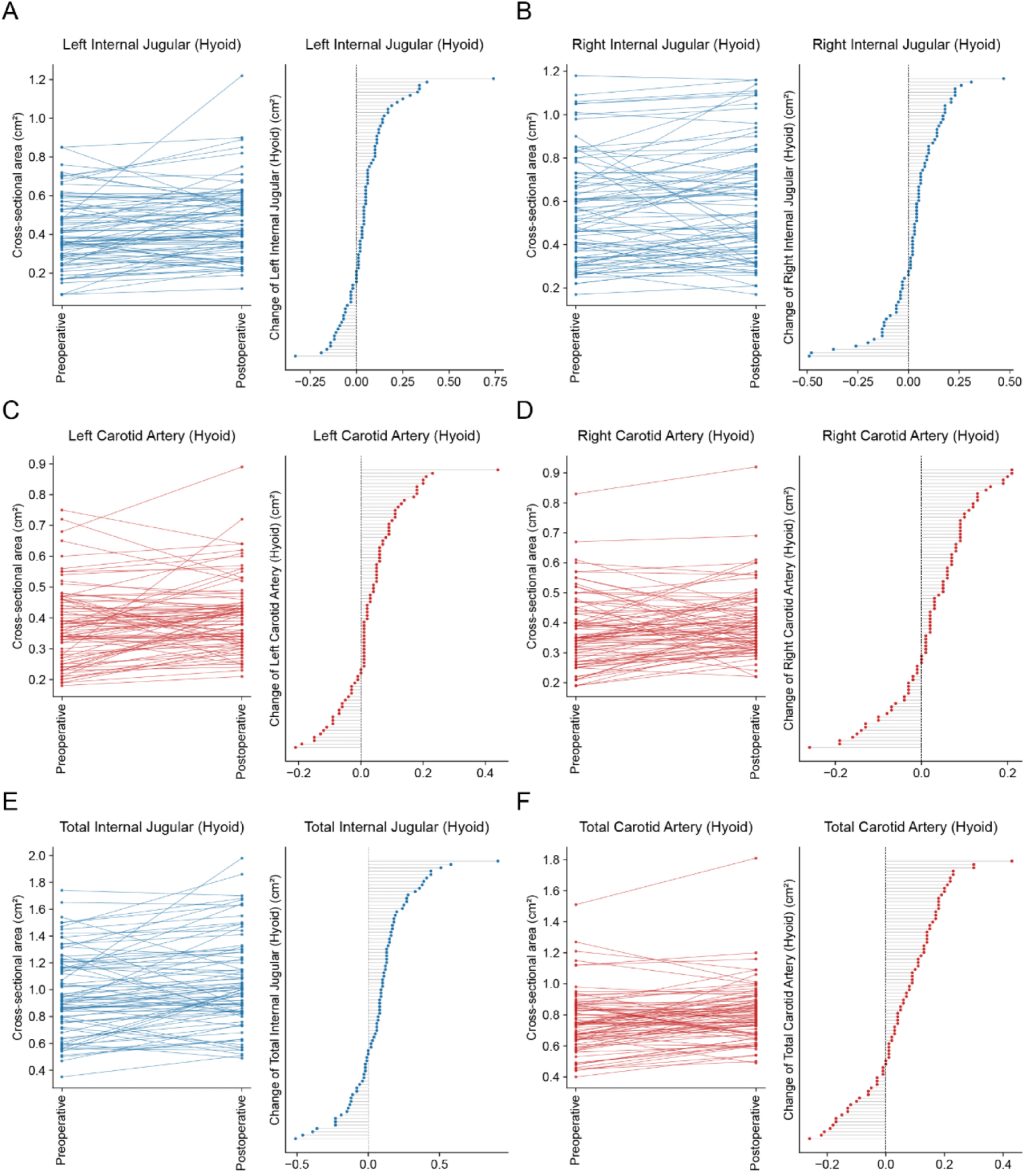
Preoperative and postoperative changes in vascular cross-sectional area at the hyoid level. (A–B) IJVs; (C–D) ICAs; (E–F) total venous and arterial cross-sectional areas. Left panels show paired pre- and postoperative measurements for each patient, connected by lines to demonstrate individual trends. Right panels depict the distribution of individual changes (postoperative minus preoperative). Blue lines represent venous structures and red lines represent arterial structures. All parameters demonstrated significant postoperative enlargement (p < 0.01; see Table 2 for quantitative results).

Quantitative evaluation demonstrated a significant increase of vascular cross-sectional areas in both veins and arteries at the hyoid level (Figure 3, Table 2).

**Table 2.** Preoperative and postoperative comparison of vascular cross-sectional areas at the hyoid level in 83 patients.

Blood vessel	Level	Side	Preoperative (cm^2^)	Postoperative (cm^2^)	Difference (cm^2^) (Post-Pre)	p
Jugular vein	hyoid	Left	0.41 ± 0.18	0.46 ± 0.19	**0.05** **±** **0.14**	0.0009
Jugular vein	hyoid	Right	0.56 ± 0.25	0.59 ± 0.26	**0.03** ± **0.15**	0.0054
Jugular vein	hyoid	Total	0.97 ± 0.31	1.05 ± 0.34	**0.08** ± **0.22**	0.0003
Carotid artery	hyoid	Left	0.38 ± 0.12	0.41 ± 0.12	**0.03** ± **0.1**	0.0024
Carotid artery	hyoid	Right	0.38 ± 0.12	0.4 ± 0.11	**0.03** ± **0.09**	0.0049
Carotid artery	hyoid	Total	0.75 ± 0.19	0.81 ± 0.19	**0.06** ± **0.13**	0.0002

At this level, the IJV was generally dilated after surgery. Specifically, in the left IJV area, 57 patients (68.67%) showed an increase, 22 (26.51%) showed a decrease, and 4 (4.82%) remained unchanged. The mean cross-sectional area increased from 0.41 cm^2^ to 0.46 cm^2^ (p = 0.0009). On the right side, 57 patients (68.67%) showed an increase, 24 (28.92%) a decrease, and 2 (2.41%) remained unchanged, with the mean area increasing from 0.56 cm^2^ to 0.59 cm^2^ (p = 0.0054). Overall, the total jugular vein cross-sectional area increased in 57 patients (68.67%), decreased in 26 (31.33%), with a mean increase from 0.97 cm^2^ to 1.05 cm^2^ (p = 0.0003).

Simultaneously, the ICA also showed a postoperative increase. On the left side, 59 patients (71.08%) showed an increase, 22 (26.51%) a decrease, and 2 (2.41%) remained unchanged, with the mean area increasing from 0.38 cm^2^ to 0.41cm^2^ (p = 0.0024). On the right side, 55 patients (66.27%) showed an increase, 25 (30.12%) a decrease, and 3 (3.61%) remained unchanged, with the mean area increasing from 0.38 cm^2^ to 0.40 cm^2^ (p = 0.0049). Overall, the total carotid artery area increased in 60 patients (72.29%), decreased in 22 (26.5%), and remained unchanged in 1 (1.20%), with the mean area increasing from 0.75 cm^2^ to 0.81 cm^2^ (p = 0.0002).

All these findings indicate that both venous and arterial luminal diameters increased after surgery, suggesting that clearance of perivascular tissues within the carotid sheath during surgery may have relieved external compression on the vessels, thereby expanding the luminal area and potentially improving regional hemodynamics. However, it was observed that the cross-sectional area of blood vessels in some patients decreased, which may be related to individual differences in vascular structure, sympathetic nerve reflexes or other vascular regulatory mechanisms.

### Postoperative changes in cervical arterial and venous blood flow

**Figure 4.**
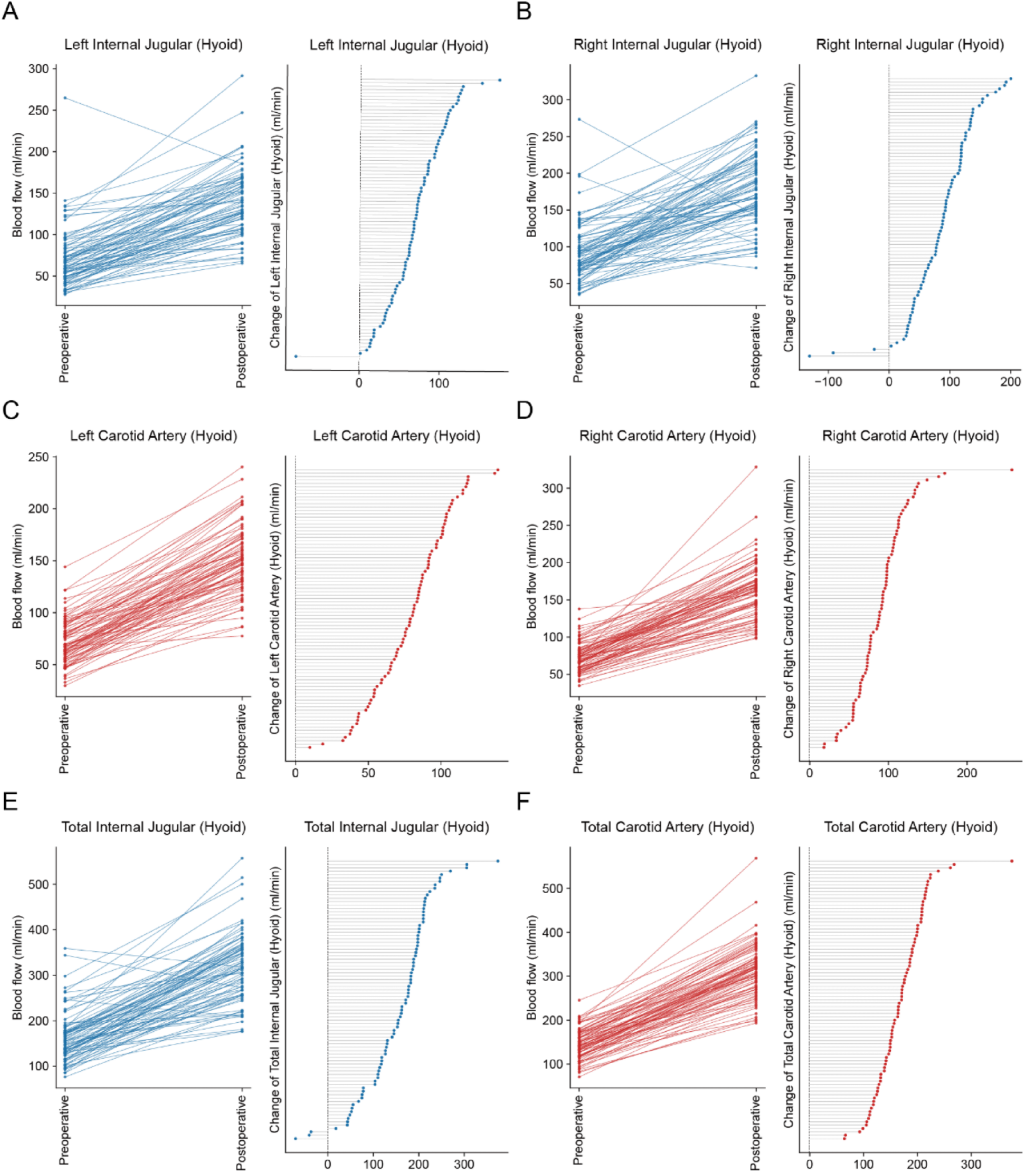
Preoperative and postoperative blood flow changes at the hyoid level. (A–B) IJVs; (C–D) ICAs; (E–F) total venous and arterial flows. Left panels depict paired pre- and postoperative flow values for each patient; right panels show the distribution of individual changes (postoperative—preoperative). Blue lines represent venous structures, and red lines represent arterial structures. All parameters demonstrated significant postoperative increases (p < 0.0001; see Table 3 for quantitative data).

**Figure 5.**
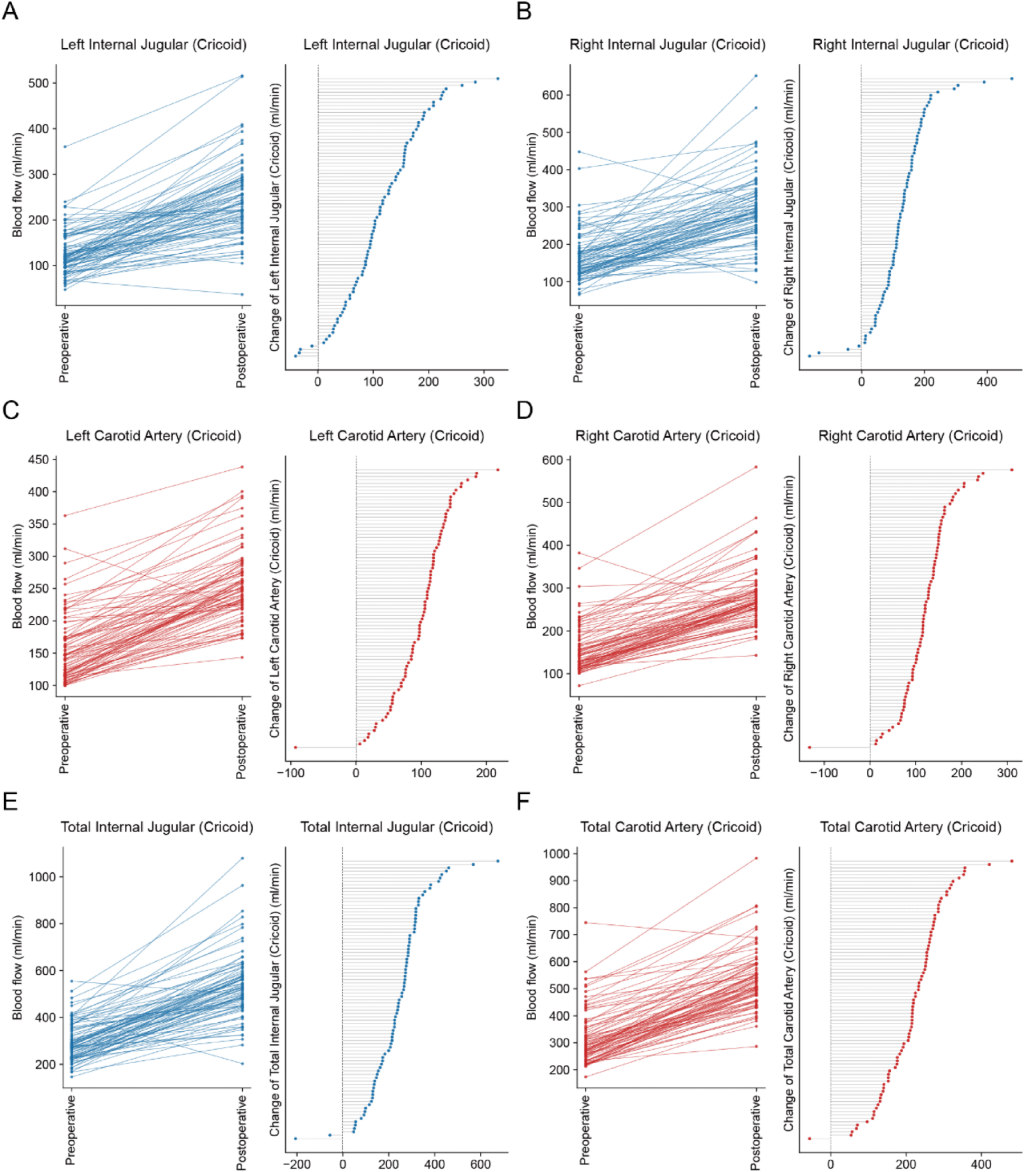
Preoperative and postoperative blood flow changes at the cricoid level. (A–B) IJVs; (C–D) CCAs; (E–F) total venous and arterial flows. Blue and red curves indicate venous and arterial structures, respectively, demonstrating parallel postoperative flow enhancement across all patients (p < 0.0001; see Table 3).

Quantitative evaluation demonstrated a significant postoperative increase in both venous and arterial blood flow at the hyoid and cervical level (Figures 4 and 5, Table 3).

**Table 3.** Preoperative and postoperative comparisons of cervical arterial and venous blood flow (ml/min) at the hyoid and cricoid levels in 83 patients.

Blood vessel	Level	Side	Preoperative (ml/min)	Postoperative (ml/min)	Difference (ml/min) (Post-Pre)	p
Jugular vein	hyoid	Left	70.2 ± 34.91	139.79 ± 40.27	**69.59** **±** **38.1**	0.0000
Jugular vein	hyoid	Right	92.04 ± 39.8	177.76 ± 50.06	**85.72** ± **55.51**	0.0000
Jugular vein	hyoid	Total	162.24 ± 55.54	317.55 ± 75.34	**155.31** ± **77.0**	0.0000
Carotid artery	hyoid	Left	71.9 ± 21.66	150.85 ± 32.41	**78.95** ± **26.46**	0.0000
Carotid artery	hyoid	Right	72.57 ± 19.8	162.83 ± 38.2	**90.26** ± **35.74**	0.0000
Carotid artery	hyoid	Total	144.46 ± 33.91	313.67 ± 60.54	**169.21** ± **47.4**	0.0000
Jugular vein	cricoid	Left	127.62 ± 50.54	242.24 ± 82.04	**114.62** ± **71.93**	0.0000
Jugular vein	cricoid	Right	167.28 ± 66.36	292.19 ± 91.59	**124.91** ± **91.43**	0.0000
Jugular vein	cricoid	Total	294.9 ± 84.12	534.44 ± 141.86	**239.54** ± **126.8**	0.0000
Carotid artery	cricoid	Left	157.22 ± 52.81	255.81 ± 56.14	**98.58** ± **46.9**	0.0000
Carotid artery	cricoid	Right	158.14 ± 56.27	278.92 ± 67.88	**120.77** ± **58.67**	0.0000
Carotid artery	cricoid	Total	315.37 ± 101.65	534.72 ± 109.69	**219.35** ± **85.38**	0.0000

### Hyoid level

At the hyoid level, blood flow in both the IJVs and ICAs increased significantly after surgery (Figure 4). The total venous flow increased in 80 patients (96.39%) and decreased in 3 (3.61%), rising from 162.24 ml/min to 317.55 ml/min (p < 0.0001). The mean flow of the left IJV increased from 70.2 ml/min to 139.79 ml/min and for the right side, from 92.04 ml/min to 177.76 ml/min (p < 0.0001).

A similar trend was observed in the ICAs, with total carotid flow increasing from 144.46 ml/min to 313.67 ml/min (p < 0.0001). The left carotid artery flow rose from 71.9 ml/min to 150.85 ml/min, and the right side from 72.57 ml/min to 162.83 ml/min.

### Cricoid level

At the cricoid level, postoperative blood flow was also improved (Figure 5). Total IJV flow increased from 294.9 ml/min to 534.44 ml/min (p < 0.0001). The left jugular vein increased from 127.62 ml/min to 242.24 ml/min, and the right side from 167.28 ml/min to 292.19 ml/min.

The CCAs also showed comparable postoperative changes. Total carotid flow increased from 315.37 ml/min to 534.72 ml/min (p < 0.0001). The left carotid artery increased from 157.22 ml/min to 255.81 ml/min, and the right side from 158.14 ml/min to 278.92 ml/min.

Across all measurements, both venous and arterial blood flow showed significant and postoperative enhancement. The simultaneous improvement of jugular vein and carotid artery flow suggests that the surgery may have influenced vascular patency and improved regional hemodynamics.

### Longitudinal ultrasound follow-up

A subgroup of nine patients underwent repeat cervical Doppler ultrasound during follow-up (Supplemental Tables 3 and 4, Supplemental Figures 2 and 3).

Vascular cross-sectional area, which expanded acutely immediately after surgery, tended to return toward or slightly below preoperative levels at follow-up in some segments. In contrast, arterial and venous blood flow not only remained elevated compared with baseline but in several segments continued to increase over time.

These findings suggest that although acute morphological dilation may partially regress, functional hemodynamic improvement appears to persist and may reflect progressive adaptation rather than transient decompression alone.

Given the small follow-up sample size (n = 9), these results should be interpreted cautiously and require validation in larger prospective longitudinal cohorts.

### Correlation between vascular cross-sectional area and blood flow at the hyoid level

Spearman correlation analyses were performed to investigate the relationship between vascular cross-sectional area and blood flow velocity at the hyoid level in both preoperative and postoperative states, as well as the correlation between their postoperative-to-preoperative differences (Table 4).

**Table 4.** Spearman correlation coefficients between vascular cross-sectional area and blood flow velocity at the hyoid level before and after surgery in 83 patients.

Blood vessel	Level	Side	Preoperative	Postoperative	Difference
Jugular Vein	Hyoid	Left	**0** **.** 42****	**0**.37***	−0.16
Jugular Vein	Hyoid	Right	0.16	0.12	−0.09
Jugular Vein	Hyoid	Total	0.09	**0**.27*	−0.15
Carotid Artery	Hyoid	Left	0.10	0.14	0.13
Carotid Artery	Hyoid	Right	−0.13	0.12	0.23 *
Carotid Artery	Hyoid	Total	−0.08	0.15	0.16

### Venous correlations

A statistically significant positive correlation was observed between the cross-sectional area and flow velocity of the left IJV both preoperatively and postoperatively, with Spearman's correlation coefficients of r = 0.42 (p < 0.0001) and r = 0.37 (p < 0.001). The total IJV also demonstrated a significant postoperative correlation r = 0.27 (p < 0.05). Although these findings seem to indicate a moderate and consistent association between venous caliber and blood flow, the clinical relevance appears limited, as no significant correlations were identified between the change in blood flow and the corresponding change in cross-sectional area, suggesting that the increase in venous drainage capacity was not directly proportional to vessel dilation.

### Arterial correlations

For the ICAs, the correlation analysis revealed that only the right carotid artery showed a statistically significant postoperative correlation between blood flow and cross-sectional area (r = 0.23, p < 0.05). However, this relationship was weak (r < 0.3). Additionally, no significant correlations were observed between changes in arterial flow velocity change and cross-sectional area change, which indicates limited clinical relevance.

The measurement results showed that the changes in blood flow velocity had no obvious relationship with vascular morphology, which may be because blood flow velocity is mainly affected by comprehensive hemodynamic factors rather than solely determined by vascular shape.

### Correlation between cervical vascular flow and neuropsychological test scores

Spearman correlation analyses were performed to examine the associations between cervical vascular blood flow and neuropsychological performance, including MMSE, MoCA, ADL, and ABS (Table 5A-D).

**Table 5.** Correlation analysis of cervical vascular flow with neuropsychological scores in 83 patients. (A) MMSE, (B) MoCA, (C) ADL, (D) ABS.

(A) MMSE						
Score	Blood Vessel	Level	Side	Preoperative	Postoperative	Difference
MMSE	Jugular Vein	Hyoid	Left	0.09	0.19	0.08
	Jugular Vein	Hyoid	Right	0.05	0.04	0.05
	Jugular Vein	Hyoid	Total	0.11	0.12	0.05
	Carotid Artery	Hyoid	Left	0.21	0.15	−0.14
	Carotid Artery	Hyoid	Right	**0** **.** 26*	0.16	−0.03
	Carotid Artery	Hyoid	Total	**0**.31**	0.20	−0.10
	Jugular Vein	Cricoid	Left	0.15	0.15	0.17
	Jugular Vein	Cricoid	Right	0.08	0.00	0.04
	Jugular Vein	Cricoid	Total	0.17	0.07	0.16
	Carotid Artery	Cricoid	Left	**0**.30**	**0**.43****	0.21
	Carotid Artery	Cricoid	Right	**0**.28*	**0**.24*	0.11
	Carotid Artery	Cricoid	Total	**0**.31**	**0**.37***	0.19

(B) MoCA						
Score	Blood Vessel	Level	Side	Preoperative	Postoperative	Difference
MoCA	Jugular Vein	Hyoid	Left	0.03	0.15	0.00
	Jugular Vein	Hyoid	Right	−0.07	0.16	−0.01
	Jugular Vein	Hyoid	Total	−0.04	0.18	−0.03
	Carotid Artery	Hyoid	Left	**0** **.** 22*	0.15	0.06
	Carotid Artery	Hyoid	Right	0.20	0.13	−0.06
	Carotid Artery	Hyoid	Total	**0**.28*	0.18	0.02
	Jugular Vein	Cricoid	Left	0.06	0.09	−0.02
	Jugular Vein	Cricoid	Right	0.07	0.02	−0.15
	Jugular Vein	Cricoid	Total	0.06	0.04	−0.07
	Carotid Artery	Cricoid	Left	0.21	**0**.33**	0.04
	Carotid Artery	Cricoid	Right	0.20	0.19	−0.12
	Carotid Artery	Cricoid	Total	**0**.22*	**0**.30**	−0.08

(C) ADL						
Score	Blood Vessel	Level	Side	Preoperative	Postoperative	Difference
ADL	Jugular Vein	Hyoid	Left	0.12	0.14	−0.06
	Jugular Vein	Hyoid	Right	0.14	0.12	−0.10
	Jugular Vein	Hyoid	Total	0.18	0.16	−0.06
	Carotid Artery	Hyoid	Left	0.21	0.14	−0.08
	Carotid Artery	Hyoid	Right	**0** **.** 35**	**0**.28**	−0.11
	Carotid Artery	Hyoid	Total	**0**.36***	**0**.28**	−0.13
	Jugular Vein	Cricoid	Left	0.05	0.02	−0.09
	Jugular Vein	Cricoid	Right	0.10	0.00	0.02
	Jugular Vein	Cricoid	Total	0.11	0.02	−0.02
	Carotid Artery	Cricoid	Left	**0**.26*	**0**.28**	0.00
	Carotid Artery	Cricoid	Right	**0**.25*	**0**.27*	−0.04
	Carotid Artery	Cricoid	Total	**0**.29**	**0**.29**	−0.02

(D) ABS						
Score	Blood Vessel	Level	Side	Preoperative	Postoperative	Difference
ABS	Jugular Vein	Hyoid	Left	0.01	−0.12	**0** **.** 22*
	Jugular Vein	Hyoid	Right	0.08	−0.11	0.08
	Jugular Vein	Hyoid	Total	0.07	−0.12	0.12
	Carotid Artery	Hyoid	Left	**0**.24*	−0.13	**0**.30**
	Carotid Artery	Hyoid	Right	0.11	0.04	−0.02
	Carotid Artery	Hyoid	Total	**0**.22*	−0.05	0.17
	Jugular Vein	Cricoid	Left	−0.02	0.02	**0**.33**
	Jugular Vein	Cricoid	Right	0.03	−0.03	0.12
	Jugular Vein	Cricoid	Total	0.02	−0.02	**0**.27*
	Carotid Artery	Cricoid	Left	−0.02	−0.05	0.12
	Carotid Artery	Cricoid	Right	0.06	−0.15	0.12
	Carotid Artery	Cricoid	Total	0.01	−0.07	0.17

### MMSE correlations

The blood flow in the **IJVs** showed weak and statistically insignificant correlations with MMSE scores at both the hyoid and cricoid levels, preoperatively and postoperatively, as well as in the changes between them. This indicates that alterations in venous outflow had a limited influence on MMSE performance.

In contrast, the carotid arteries exhibited moderate positive correlations with MMSE scores. At the hyoid level, preoperative (*r* = 0.31, *p* < 0.01) total carotid flow were correlated with MMSE scores. Similarly, at the cricoid level, at the cricoid level, both preoperative (*r* = 0.31, *p* < 0.01) and postoperative (*r* = 0.37, *p* < 0.001) total carotid flow also showed a significant correlation. These results suggest that higher carotid flow may be associated with higher MMSE scores. However, no significant correlation was found between the improvement in cognitive function and the increase in carotid blood flow.

### MoCA correlations

Similar trends were observed in the correlations with MoCA scores. Carotid flow exhibited mild to moderate positive correlations with MoCA preoperatively at the hyoid level (*r* = 0.28, *p* < 0.05) and at the cricoid level (*r* = 0.22, *p* < 0.05), as well as postoperatively at the cricoid level (*r* = 0.30, *p* < 0.01). In contrast, venous flow showed weak and nonsignificant correlations. No significant correlations were found between MoCA score changes and blood flow changes.

### ADL correlations

A comparable pattern was observed for ADL scores. At the hyoid level, total carotid flow showed significant correlations with ADL scores both preoperatively (r = 0.36, p < 0.001) and postoperatively (r = 0.28, p < 0.01). Similarly, at the cricoid level, significant correlations were observed preoperatively (r = 0.29, p < 0.01) and postoperatively (r = 0.29, p < 0.01). However, venous flow again showed no meaningful relationship with ADL scores. Furthermore, changes in ADL scores were not significantly related to changes in blood flow.

### ABS correlations

In contrast to the other cognitive measures, the ABS scores showed a distinct pattern. Almost no significant correlations with agitation severity were observed with preoperative and postoperative arterial or venous blood flow. However, flow changes, particularly in left-sided vessels, were significantly correlated with reductions in ABS scores, since correlations were observed in both arterial and venous flows (hyoid level vein: r = 0.22, p < 0.05; hyoid level artery: r = 0.30, p < 0.01; cricoid level vein: r = 0.33, p < 0.01). These findings suggest that blood flow velocity alone is not significantly related to behavioral agitation, whereas improvements in flow dynamics may contribute to greater behavioral stability.

Overall, these results highlight the correlation between cervical vascular hemodynamics and cognitive, functional, and behavioral outcomes. Blood flow velocity, particularly carotid artery flow, showed consistent positive correlations with cognitive and functional measures (MMSE, MoCA, and ADL), while behavioral performance (ABS) was influenced more by hemodynamic changes than by absolute blood flow velocity. These findings suggest that surgery may has contribute to improvements in cognitive and behavioral performance but cannot determine the extent of the improvement.

### Safety

Across the cohort of 83 patients, no major complications, systemic morbidity, reoperation, or procedure-related mortality were observed. Minor postoperative events were documented, all of which were classified as Clavien–Dindo grade I–II. Wound pain was the most common issue, occurring in 26 patients and managed effectively with oral analgesics. Delayed wound healing was observed in 3 patients (Cases #9, #26, and #83), all of whom achieved complete recovery after local wound care. Emotional fluctuation occurred in 9 patients, while sleep disturbance was reported in 3 patients; both conditions improved with supportive management. Overall, all postoperative adverse events were minor and resolved without the need for invasive intervention. A detailed summary of adverse events is provided in Supplemental Table 1.

## Discussion

This study describes significant postoperative changes in cervical hemodynamics in patients with AD. Postoperative assessments revealed significant increases in arterial and venous flow at the level of the hyoid bone and cricoid cartilage. These hemodynamic changes were associated with improvements in overall cognition, executive function, activities of daily living, and behavioral regulation. However, given the observational design of this study, these findings should be interpreted as associative rather than causal.

Correlation analyses further demonstrated that CCA and ICA blood flow were positively correlated with cognitive and functional recovery, whereas venous flow showed weaker or nonsignificant correlations. Additionally, little correlation was observed between vascular cross-sectional area and flow velocity, with a significant correlation only in the left IJV, suggesting a complex coupling between vessel morphology and hemodynamics.

From a cerebrovascular perspective, the observed postoperative increase in carotid arterial flow is of particular interest. Impaired arterial inflow and chronic cerebral hypoperfusion are well-recognized contributors to cognitive decline in AD. In this context, the improvement in carotid flow observed in our study, together with its correlation with cognitive and functional measures, suggests that enhanced extracranial arterial hemodynamics may represent an important contributor to the postoperative changes. Therefore, the findings of the present study may be interpreted within a broader cerebrovascular framework, in which surgical modification of the cervical compartment influences vascular hemodynamics rather than acting through a purely lymphatic mechanism.

Together, these findings indicate that the surgical procedure, including decompression and tissue manipulation, may have contributed to extravascular compression within the carotid sheath, restored cervical blood flow, altered hemodynamics following the surgery.

### Restoration of cervical hemodynamics

The consistent postoperative enlargement of both carotid and jugular vessels observed in this study indicates that dcLVA effectively relieves extrinsic compression within the carotid sheath. Given that cerebral hypoperfusion is an early and potentially reversible component of AD pathophysiology, interventions aimed at optimizing extracranial hemodynamics may hold therapeutic promise, particularly in patients with imaging evidence of cervical vascular narrowing or impaired venous outflow.

From a hemodynamic standpoint, enlargement of the vascular cross-sectional area generally leads to reduced flow velocity, which may decrease volumetric flow if compensatory mechanisms are not activated. Arterial flow regulation is primarily governed by intrinsic neural and cardiac control, reflecting the arteries’ autoregulatory capacity to maintain perfusion pressure. In contrast, venous flow is largely dependent on extrinsic factors such as respiratory variation, muscle contraction, and venous valves, making it more susceptible to external compression and less capable of self-regulation.

Anatomical asymmetries may also contribute to these findings. The CCA bifurcates into internal and external branches, whereas the IJV follows a single, unbranched trajectory. Furthermore, baseline venous drainage is characteristically asymmetric, with the left IJV typically smaller and exhibiting greater variability than the right.

### Mechanistic interpretation

The carotid sheath is a confined fascial compartment that encloses the CCA/ICA, IJV, vagus nerve, and deep cervical lymph nodes embedded within adipose and connective tissue.^14–16^ Imaging and anatomical studies show that lymph nodes and fatty connective tissue within this space can alter vascular geometry or exert compressive effects, thereby restricting vascular expandability.

Previous case reports document IJV stenosis caused by compression from a tortuous ICA, and dense lymphatic tissue producing >90% stenosis of the ICA, both with restoration of luminal diameter and flow after surgical removal of the compressive tissue.^17,18^ In the context of LVA/dcLVA, intraoperative clearance of perivascular lymph nodes, adipose tissue, and connective septa within the carotid sheath likely releases external compression on both the IJV and the carotid artery, enhancing vascular compliance and luminal expandability. The resulting reduction in perivascular resistance provides a plausible explanation for the significant postoperative increases in cross-sectional area and flow observed in this cohort, consistent with the anatomical and clinical literature above. Because this was a single-center observational study without a control group, the specific contribution of individual surgical components cannot be definitively determined. Opening of the carotid sheath, removal of perivascular lymphatic and adipose tissue, autonomic modulation related to adventitial manipulation, and LVA may all contribute to the observed hemodynamic changes. Therefore, the present findings should be interpreted as associative rather than causal, and future controlled studies comparing decompression alone with decompression plus LVA will be required.

However, a small number of patients experienced a decrease in vascular cross-sectional area after surgery. This seemingly contradictory result may be caused by multiple factors. Increased sympathetic nerve activity, local inflammatory edema, or changes in venous return pressure after surgery may all cause temporary vascular constriction. In addition, blood vessels that have been under long-term pressure may undergo intramural fibrosis or structural remodeling in the chronic stage, resulting in reduced elasticity, making it difficult for the blood vessels to fully expand even when the external compression is released.^19–22^ Further longitudinal evaluation is needed to determine whether these changes represent transient physiological adaptations or fixed structural features of the vascular wall. Nonetheless, the blood flow velocity in the vast majority of patients improved significantly after surgery, supporting the hemodynamic benefit of surgical decompression.

It should be emphasized that this study did not directly measure cervical or cerebral lymphatic flow. Owing to the absence of clinically validated techniques for quantifying deep cervical lymphatic flow or compartmental pressure, vascular hemodynamic parameters were used as indirect indicators of changes within the lymphatic-vascular environment of the neck. Consequently, any inference regarding lymphatic drainage or clearance should be interpreted with caution and viewed as hypothesis-generating rather than definitive evidence.

### Functional and cognitive recovery, and clinical implications

The concomitant improvement in cognitive, functional, and behavioral outcomes following dcLVA underscores the potential neurological benefits of restoring physiological vascular dynamics. The moderate correlations between carotid flow and MMSE, MoCA, and ADL scores support the notion that enhancing arterial supply contributes to improved neural activity. In advanced AD, global cognitive screening instruments (e.g., MMSE and MoCA) are subject to marked floor effects and limited responsiveness. Therefore, functional (ADL) and behavioral (ABS) improvements may better capture clinically meaningful changes in this severe-stage cohort.

The present findings provide novel insight into the clinical significance of cervical vascular decompression in the management of AD. The observed improvements in arterial inflow, venous drainage, and neuropsychological function suggest that dcLVA may serve as a potential adjunctive intervention for AD patients with demonstrable cervical vascular compromise.

### Comparison with previous studies

A substantial body of work has linked cerebral hypoperfusion and impaired extracranial hemodynamics with AD. Systematic reviews and meta-analyses report reduced cerebral blood flow in AD and, to a lesser extent, in mild cognitive impairment, with positive associations between regional perfusion and cognition.^15,23^ Our preoperative findings of reduced carotid and jugular flows align with these literatures and suggest that cervical vascular compromise contributes to the perfusion deficit commonly observed in AD.

What differentiates the present study is that postoperative surgical decompression (via LVA/dcLVA) produced parallel improvements in arterial inflow and venous outflow together with measurable gains in cognition and daily functioning, which is rarely demonstrated in prior work that focused on non-surgical strategies. This mechanical approach restores cervical vascular patency by relieving external compression rather than addressing endovascular pathology and therefore may serve as a supplement to, rather than duplication of, conventional endovascular or medical therapy.

Additionally, our findings are congruent with the broader literature on jugular venous outflow obstruction and styloidogenic/cervical spondylotic IJV compression, which has implicated extrinsic anatomic constraints in altered cranial venous drainage and related neurological symptoms. Surgical or structural relief of such constraints has been associated with hemodynamic normalization,^24^ conceptually paralleling the decompressive effect achieved in our cohort.

### Potential role in multimodal treatment

DcLVA should not be regarded as a stand-alone cure for AD but rather as a complementary approach targeting a modifiable peripheral factor, cervical vascular resistance. In clinical practice, dcLVA could potentially be integrated into multimodal management strategies, alongside pharmacologic and cognitive interventions, to improve cerebral perfusion and delay functional decline. The procedure's minimally invasive nature, combined with its measurable hemodynamic benefits, supports its feasibility for selected patients with coexisting vascular or lymphatic abnormalities.

### Limitations and future directions

Despite its encouraging results, this study has several limitations that warrant consideration.

First, it was conducted as a single-center observational study with a limited sample size and without a randomized control group. Future randomized or multicenter controlled trials are needed to validate these preliminary findings and to exclude potential confounding factors, such as placebo effects or natural disease fluctuation. Because this study includes multiple surgical components (carotid sheath opening, tissue removal, and lymphatic–venous anastomosis), the independent effect of dcLVA cannot be isolated. It should be clarified that the surgical procedure described in this study involves decompression of the deep cervical lymphatic-vascular compartment rather than cervical spine decompression. Opening of the carotid sheath and removal of perivascular tissue may play a major role in the observed hemodynamic changes. LVA is intended to provide a potential long-term lymphatic outflow pathway; however, the present observational design without a decompression-only control group does not allow definitive separation of the effects of decompression and anastomosis. Future controlled studies are required to address this question.

Second, although postoperative hemodynamic improvements were objectively confirmed by Doppler ultrasonography, the absence of direct cerebral perfusion imaging (e.g., arterial spin labeling MRI, CT perfusion) limits our ability to quantify intracranial blood flow changes. Biomarkers related to Aβ pathology, CSF dynamics, or cerebral lymphatic drainage were not evaluated. Incorporating multimodal imaging modalities in future studies would enable a more precise assessment of the link between cervical vascular decompression and cortical perfusion dynamics.

Third, although longitudinal neuropsychological follow-up demonstrated sustained improvement up to 9 months and vascular flow enhancement persisted in a subgroup of patients, the follow-up duration remains limited and the ultrasound subgroup was small. Longer-term prospective observation with larger cohorts will be necessary to confirm durability beyond the first postoperative year. It should be emphasized that the observed postoperative changes do not indicate reversal of neurodegeneration. Rather, they likely reflect functional modulation related to autonomic regulation and improved cerebral perfusion in a severely compromised neural system.

Additionally, the heterogeneity of AD pathology poses additional complexity. While the present results suggest that patients with pronounced vascular compromise may derive greater benefit from dcLVA, future work should aim to stratify patients by disease stage, vascular phenotype, and CSF or amyloid biomarker profiles to better define the target population for this intervention.

Further mechanistic studies, including cerebrovascular reactivity testing, lymphatic imaging, and neurovascular coupling assessment, will be necessary to elucidate the biological pathways by which peripheral hemodynamic restoration influences central neural function.

### Conclusion

This study demonstrates postoperative changes in cervical vascular hemodynamics and clinical outcomes in patients with AD. Due to the observational design and lack of a control group, the specific contribution of dcLVA cannot be determined. The findings should therefore be interpreted as descriptive and hypothesis-generating.

The findings support the concept that extracranial vascular compression and impaired lymphatic drainage may contribute to cerebral hypoperfusion and cognitive decline in AD, and that surgical decompression of the carotid sheath can partially reverse these changes. By addressing the peripheral vascular and lymphatic components of cerebral circulation, dcLVA offers a novel, minimally invasive approach to improving brain perfusion and function.

While further large-scale, controlled, and multimodal studies are required to confirm these results, the present work introduces a promising new avenue for managing the vascular component of neurodegenerative disease and broadens the therapeutic perspective for AD beyond traditional neurocentric paradigms.

## Supplemental Material

sj-docx-1-alz-10.1177_13872877261459037 - Supplemental material for Postoperative changes in cervical hemodynamics and cognitive function following cervical lymphatic-venous surgery in Alzheimer's disease Supplemental material, sj-docx-1-alz-10.1177_13872877261459037 for Postoperative changes in cervical hemodynamics and cognitive function following cervical lymphatic-venous surgery in Alzheimer's disease by Xiaoju Zheng, Cong Tang, Haijun Li, Baoshan Wang, Yuqi Zheng, Wenbin Song, Aimei Wu, Linjun Xin, Dengwen Zhang, Rongguo Yang, Shuang Du, Peng He, Yujiao Li, Linjuan Wu, Qi Shi and Gonçalo J.L. Bernardes in Journal of Alzheimer's Disease
