# Supplementary material for: Postoperative changes in cervical hemodynamics and cognitive function following cervical lymphatic-venous surgery in Alzheimer's disease
Source: J Alzheimers Dis. 2026 Jun 26;112(3):1536–51. doi: 10.1177/13872877261459037 (PMC13392209; doi:10.1177/13872877261459037)
Supplement: sj-docx-1-alz-10.1177_13872877261459037 - Supplemental material for Postoperative changes in cervical hemodynamics and cognitive function following cervical lymphatic-venous surgery in Alzheimer's disease [file sj-docx-1-alz-10.1177_13872877261459037.docx]

## **Supplemental Material**

## **Postoperative changes in cervical hemodynamics and cognitive function following cervical lymphatic-venous surgery in Alzheimer’s disease**

### Supplemental Table 1. Perioperative Adverse Events (graded by Clavien–Dindo classification)

| **Adverse Event** | **n (patients)** | **Clavien–Dindo Grade** | **Representative Cases** | **Management** | **Outcome** |
| --- | --- | --- | --- | --- | --- |
| Wound pain | 26 | I | Multiple | Oral analgesics | Resolved within weeks |
| Delayed wound healing | 3 | II | #9, #26, #83 | Dressing change, local care | Complete healing |
| Emotional fluctuation | 9 | I | #6, #8, #19, #26, #52, #62, #63, #68, #69 | Supportive care | Symptoms improved |
| Sleep disturbance | 3 | I | #71, #82, #83 | Supportive care | Improved |
| Major complications (systemic morbidity, reoperation, mortality) | 0 | – | – | – | – |

**
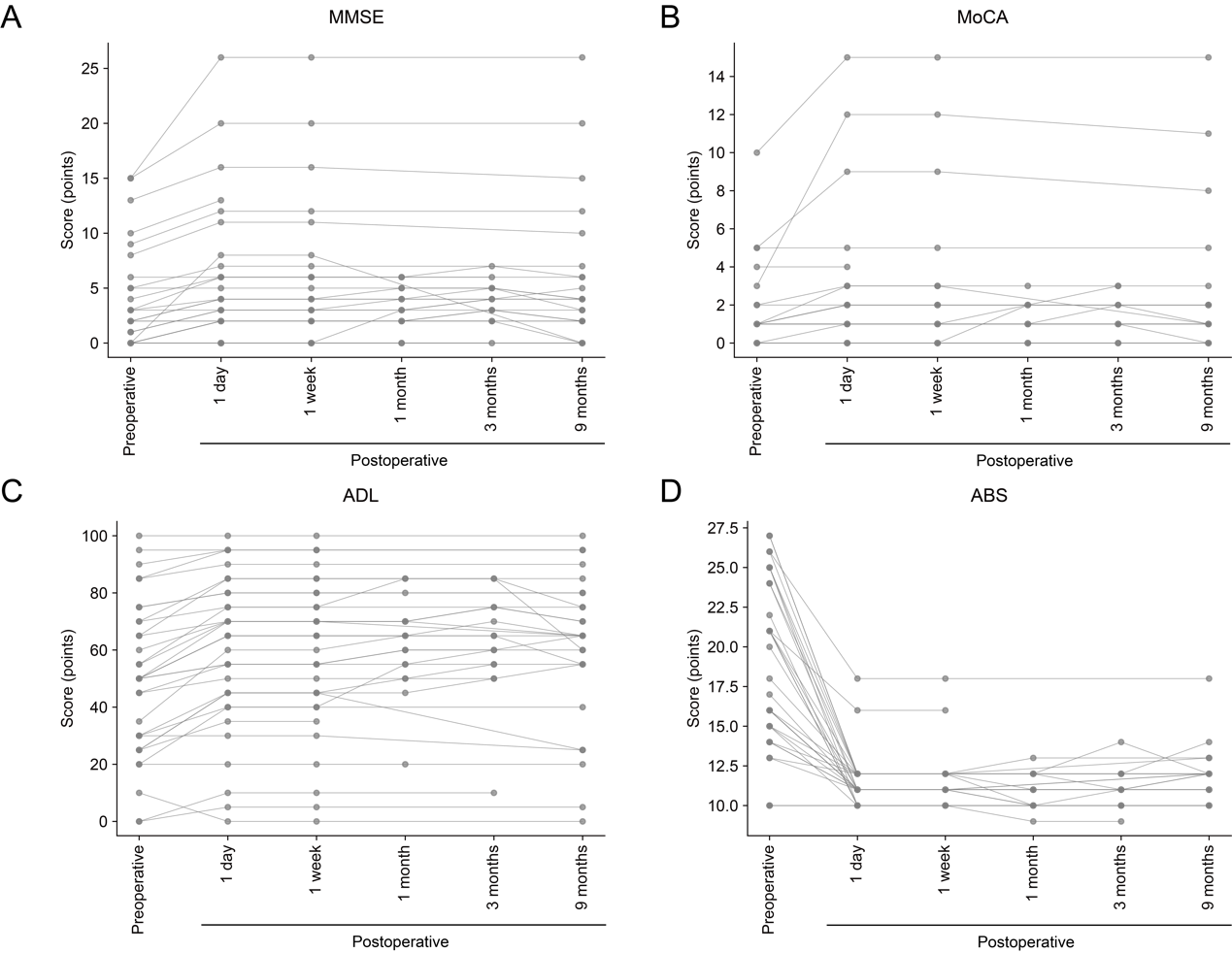
**

**Supplemental Figure 1. Preoperative, postoperative, and follow-up changes in neuropsychological and behavioral scores (n=34).** (A) Mini-Mental State Examination (MMSE), (B) Montreal Cognitive Assessment (MoCA), (C) Activities of Daily Living (ADL), and (D) Agitation Behavior Scale (ABS) scores were evaluated preoperatively and at 1 day, 1 week, 1 month, 3 months, and 9 months postoperatively. Individual patient trajectories are shown with connected lines, illustrating longitudinal changes across follow-up time points. Upward trends in MMSE, MoCA, and ADL indicate improvement in cognitive and functional performance, whereas downward trends in ABS reflect reduced agitation severity. Overall patterns demonstrate postoperative improvement in cognitive and functional measures accompanied by decreased behavioral disturbance over time. All p values represent comparisons with the preoperative baseline.

**Supplemental Table 2. Preoperative and postoperative follow-up changes in scores**

**(A) MMSE**

| Score | Surgical Phase | Time point | Mean ± SD | p |
| --- | --- | --- | --- | --- |
| MMSE | Preoperative | — | 3.21±4.48 | — |
|  | Postoperative | 1 day | 4.97±6.21 | 0.0001 |
|  |  | 1 week | 4.73±6.14 | 0.0001 |
|  |  | 1 month | 3.06±1.76 | 0.0008 |
|  |  | 3 months | 3.64±1.86 | 0.0027 |
|  |  | 9 months | 5.07±6.49 | 0.0007 |

**(B) MoCA**

| Score | Surgical Phase | Time point | Mean ± SD | p |
| --- | --- | --- | --- | --- |
| MoCA | Preoperative | — | 1.41±2.00 | — |
|  | Postoperative | 1 day | 2.18±3.40 | 0.0041 |
|  |  | 1 week | 2.12±3.43 | 0.0041 |
|  |  | 1 month | 1.11±0.83 | 0.0196 |
|  |  | 3 months | 1.29±0.91 | 0.0339 |
|  |  | 9 months | 2.33±3.56 | 0.0080 |

**(C) ADL**

| Score | Surgical Phase | Time point | Mean ± SD | p |
| --- | --- | --- | --- | --- |
| ADL | Preoperative | — | 49.56±26.38 | — |
|  | Postoperative | 1 day | 59.41±26.42 | 0.0000 |
|  |  | 1 week | 58.33±26.06 | 0.0000 |
|  |  | 1 month | 61.94±15.45 | 0.0003 |
|  |  | 3 months | 61.43±18.75 | 0.0009 |
|  |  | 9 months | 60.74±26.52 | 0.0007 |

**(D) ABS**

| Score | Surgical Phase | Time point | Mean ± SD | p |
| --- | --- | --- | --- | --- |
| ABS | Preoperative | — | 18.65±5.22 | — |
|  | Postoperative | 1 day | 11.44±1.62 | 0.0000 |
|  |  | 1 week | 11.45±1.64 | 0.0000 |
|  |  | 1 month | 11.00±1.03 | 0.0002 |
|  |  | 3 months | 11.07±1.16 | 0.0010 |
|  |  | 9 months | 11.59±1.67 | 0.0000 |


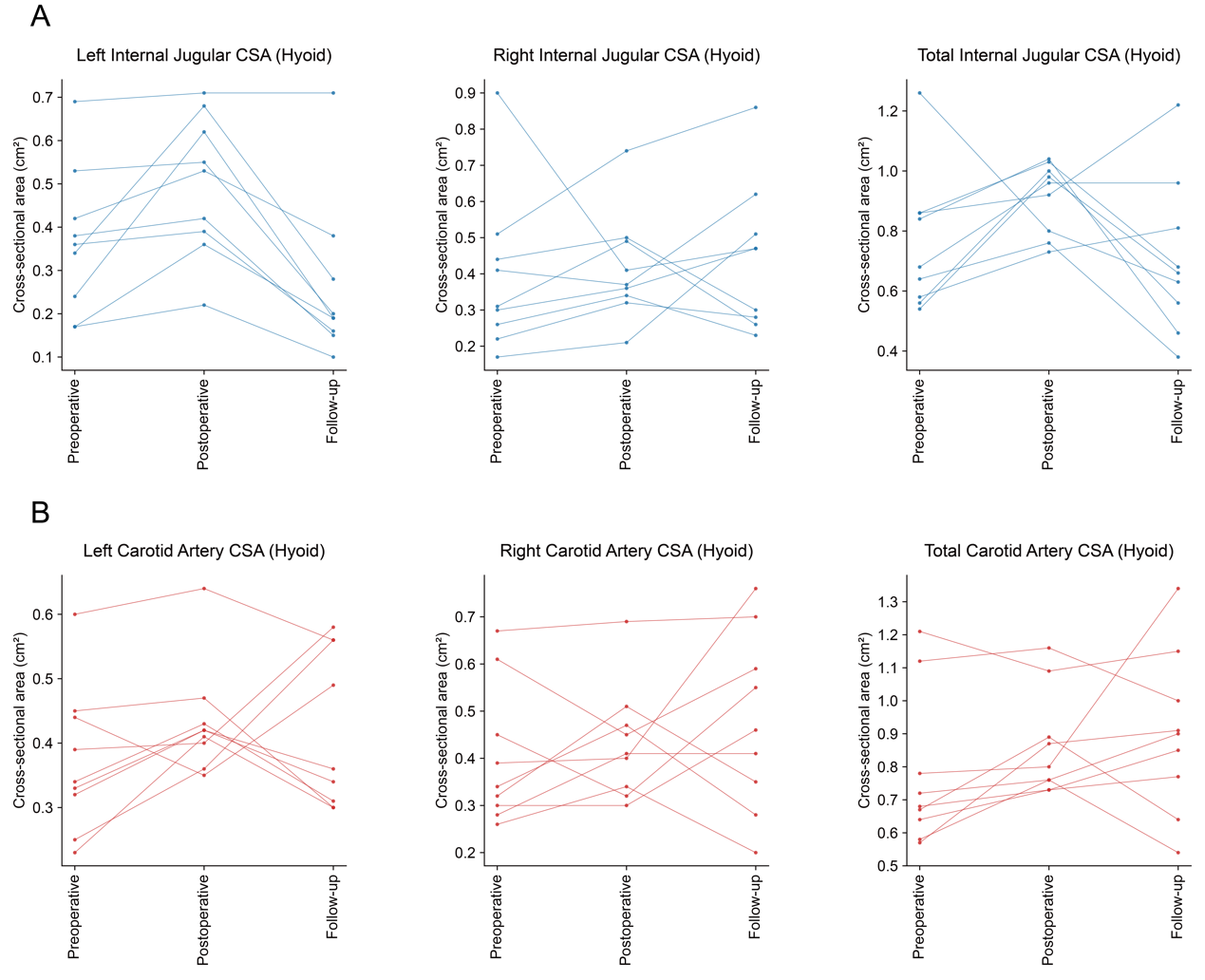


**Supplemental Figure 2. Preoperative, postoperative, and follow-up changes in vascular cross-sectional area at the hyoid level (n=9).** (A) Internal jugular vein (IJV) cross-sectional area (CSA) and (B) carotid artery (CA) cross-sectional area (CSA) were assessed preoperatively, postoperatively, and at follow-up. Within each panel, left, right, and total cross-sectional areas are shown. Individual preoperative, postoperative, and follow-up values are connected by lines, illustrating longitudinal changes across patients. Blue lines represent venous structures (IJVs), and red lines represent arterial structures (carotid arteries). Cross-sectional area is expressed in cm². All p values represent comparisons with the preoperative baseline.

**Supplemental Table 3. Preoperative, postoperative, and follow-up comparison of vascular cross-sectional areas at the hyoid level**

| Blood Vessel | Level | Side | Preoperative  (cm²) | Postoperative  (cm²) | p | Follow-up  (cm²) | p |
| --- | --- | --- | --- | --- | --- | --- | --- |
| Jugular Vein | Hyoid | Left | 0.37±0.17 | 0.50±0.16 | 0.0039 | 0.26±0.19 | 0.0195 |
| Jugular Vein | Hyoid | Right | 0.39±0.22 | 0.42±0.15 | 0.1719 | 0.44±0.21 | 0.4961 |
| Jugular Vein | Hyoid | Total | 0.76±0.23 | 0.91±0.12 | 0.1250 | 0.71±0.26 | 0.7422 |
| Carotid Artery | Hyoid | Left | 0.37±0.11 | 0.43±0.09 | 0.0508 | 0.42±0.12 | 0.3594 |
| Carotid Artery | Hyoid | Right | 0.40±0.15 | 0.43±0.12 | 0.5156 | 0.48±0.19 | 0.1562 |
| Carotid Artery | Hyoid | Total | 0.77±0.23 | 0.87±0.16 | 0.0547 | 0.90±0.25 | 0.1641 |


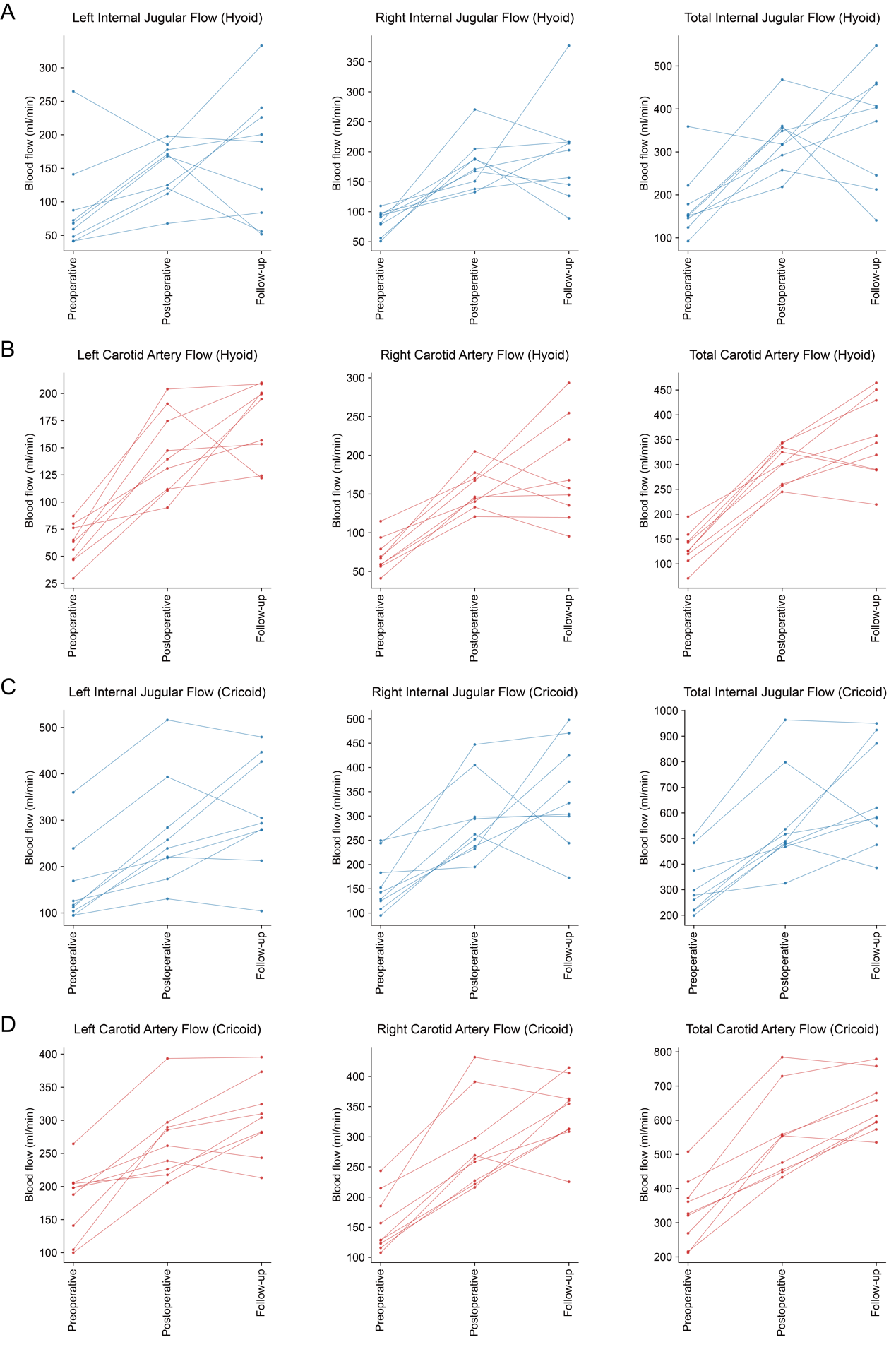


**Supplemental Figure 3. Preoperative, postoperative, and follow-up changes in vascular blood flow at the hyoid and cricoid levels (n=9).** (A) Internal jugular vein (IJV) blood flow and (B) carotid artery (CA) blood flow at the hyoid level, and (C) IJV blood flow and (D) CA blood flow at the cricoid level were assessed preoperatively, postoperatively, and at follow-up. Within each panel, left, right, and total blood flow values are shown. Individual preoperative, postoperative, and follow-up values are connected by lines, illustrating longitudinal changes across patients. Blue lines represent venous structures (IJVs), and red lines represent arterial structures (carotid arteries). Blood flow is expressed in mL/min. All p values represent comparisons with the preoperative baseline.

**Supplemental Table 4. Preoperative, postoperative, and follow-up comparison of vascular blood flow at the hyoid and cricoid levels**

| Blood Vessel | Level | Side | Preoperative  (ml/min) | Postoperative  (ml/min) | p | Follow-up  (ml/min) | p |
| --- | --- | --- | --- | --- | --- | --- | --- |
| Jugular Vein | Hyoid | Left | 91.59±71.92 | 147.22±42.95 | 0.0547 | 166.66±95.48 | 0.0117 |
| Jugular Vein | Hyoid | Right | 83.77±19.48 | 179.06±41.84 | 0.0039 | 193.91±82.41 | 0.0039 |
| Jugular Vein | Hyoid | Total | 175.36±77.38 | 326.28±70.91 | 0.0078 | 360.57±133.07 | 0.0039 |
| Carotid Artery | Hyoid | Left | 61.31±18.32 | 144.91±37.88 | 0.0039 | 174.41±35.64 | 0.0039 |
| Carotid Artery | Hyoid | Right | 71.04±22.16 | 156.07±26.10 | 0.0039 | 177.01±65.62 | 0.0039 |
| Carotid Artery | Hyoid | Total | 132.36±34.72 | 300.98±38.75 | 0.0039 | 351.42±82.80 | 0.0039 |
| Jugular Vein | Cricoid | Left | 157.53±88.87 | 270.43±117.73 | 0.0039 | 314.08±119.77 | 0.0039 |
| Jugular Vein | Cricoid | Right | 158.72±55.96 | 291.51±83.20 | 0.0039 | 345.67±106.12 | 0.0039 |
| Jugular Vein | Cricoid | Total | 316.26±115.67 | 561.94±194.69 | 0.0039 | 659.74±204.48 | 0.0039 |
| Carotid Artery | Cricoid | Left | 178.27±53.27 | 268.40±57.42 | 0.0039 | 303.03±57.61 | 0.0039 |
| Carotid Artery | Cricoid | Right | 155.93±48.04 | 286.28±76.27 | 0.0039 | 339.77±57.72 | 0.0039 |
| Carotid Artery | Cricoid | Total) | 334.20±95.63 | 554.68±125.31 | 0.0039 | 642.80±83.27 | 0.0039 |
